# The Phenotypic Characterization of the Oldest Italian Man from December 28, 2020, to September 23, 2021, A.T., Strengthens the Idea That the Immune System can Play a Key Role in the Attainment of Extreme Longevity

**DOI:** 10.3390/jcm12247591

**Published:** 2023-12-09

**Authors:** Giulia Accardi, Anna Aiello, Stefano Aprile, Anna Calabrò, Rosalia Caldarella, Calogero Caruso, Marcello Ciaccio, Francesco Dieli, Mattia Emanuela Ligotti, Serena Meraviglia, Giuseppina Candore

**Affiliations:** 1 Laboratory of Immunopathology and Immunosenescence, Department of Biomedicine, Neurosciences and Advanced Diagnostics, University of Palermo, 90133 Palermo, Italy; giulia.accardi@unipa.it (G.A.); anna.aiello@unipa.it (A.A.); anna.calabro@unipa.it (A.C.); mattiaemanuela.ligotti@unipa.it (M.E.L.); giuseppina.candore@unipa.it (G.C.); 2 Unit of Transfusion Medicine, San Giovanni di Dio Hospital, 92100 Agrigento, Italy; cot.aprile.stefano@gmail.com; 3 Department of Laboratory medicine, University Hospital “P. Giaccone”, 90127 Palermo, Italy; rosalia.caldarella@policlinico.pa.it (R.C.); marcello.ciaccio@unipa.it (M.C.); 4 Section of Clinical Biochemistry, Clinical Molecular Medicine and Clinical Laboratory Medicine, Department of Biomedicine, Neurosciences and Advanced Diagnostics, University of Palermo, 90133 Palermo, Italy; 5 Central Laboratory of Advanced Diagnosis and Biomedical Research, University Hospital “P. Giaccone”, 90127 Palermo, Italy; francesco.dieli@unipa.it (F.D.); serena.meraviglia@unipa.it (S.M.); 6 Department of Biomedicine, Neurosciences and Advanced Diagnostics, University of Palermo, 90133 Palermo, Italy

**Keywords:** ageing, ARIP, biological ageing, inflamm-ageing, INFLA score, lymphocyte subsets, longevity, semi-supercentenarian

## Abstract

In this paper, we present demographic, clinical, anamnestic, cognitive, and functional data, as well as haematological, haematochemical, immunological, and genetic parameters of an exceptional individual: A.T., a semi-supercentenarian who held the title of the oldest living Italian male centenarian from 28 December 2020, to 23 September 2021. The purpose of this study is to provide fresh insights into extreme phenotypes, with a particular focus on immune-inflammatory parameters. To the best of our knowledge, this study represents the first phenotypic investigation of a semi-supercentenarian, illustrating both INFLA-score, a metric designed to assess the cumulative impact of inflammatory markers and indicators of age-related immune phenotype (ARIP), recognized as significant gauges of biological ageing. The aim of this study was, indeed, to advance our understanding of the role of immune-inflammatory responses in achieving extreme longevity. The results of laboratory tests, as well as clinical history and interview data, when compared to the results of our recent study on Sicilian centenarians, demonstrate an excellent state of health considering his age. Consistent with previous studies, we observed increased IL-6 inflammatory markers and INFLA score in A.T. More interestingly, the semi-supercentenarian showed values of ARIP indicators such as naïve CD4+ cells, CD4+/CD8+ ratio, and CD4+TN/TM ratio in the range of young adult individuals, suggesting that his immune system’s biological age was younger than the chronological one. The results support the notion that the immune system can play a role in promoting extreme longevity. However, this does not rule out the involvement of other body systems or organs in achieving extreme longevity.

## 1. Introduction

The increasing ageing of the population presents a growing set of challenges to public health [1]. Healthcare costs in numerous countries have soared due to the increasing number of individuals facing health issues as they age, leading to a corresponding surge in severe age-related disabilities. However, laboratory experiments conducted on models have shown that ageing is not an irreversible process. In fact, interventions aimed at slowing down or postponing the ageing process and extending the period of an active life are achievable [2,3]. Therefore, gaining an understanding of the mechanisms of longevity has the potential to partially change the trajectory of the ageing process.

In this context, studying models of healthy ageing and extreme longevity holds paramount importance. Long-lived individuals (LLIs, >90), centenarians (≥100 years), semi-supercentenarians (≥105 years), and supercentenarians (≥110 years) are subjects of intense investigation. Centenarians, in particular, stand out as the most exemplary individuals in successful ageing. They have managed to withstand diseases or endure age-related conditions such as cancer, diabetes, cardiovascular disease, and stroke, making them shining examples of positive biology [4].

However, it is important to note that the increasing number of centenarians can be attributed to advancements in hygiene and sanitary conditions, as well as improvements in the quality of nutrition. These factors have enabled a greater number of older individuals to achieve exceptional longevity. Consequently, the current and future centenarians are likely to be less rigorously selected compared to those of a few decades ago [5]. 

Semi-supercentenarians and supercentenarians constitute a highly selected population, comprising individuals who have survived two World Wars along with a myriad of environmental and microbial challenges, including the Spanish flu [6,7,8,9,10]. Consequently, it is reasonable to deduce that the immune systems of these individuals possess distinct characteristics that empower them to achieve remarkable levels of human longevity. Thus, studying them can shed light on the immune-inflammatory responses that should contribute either positively or negatively to the attainment of extreme longevity [10,11].

In this paper, we present demographic, clinical, anamnestic, cognitive, and functional data, along with biochemical, genetic, and immune-inflammatory parameters of an exceptional individual: Antonino Turturici (A.T.), a semi-supercentenarian who held the title of the oldest living Italian male centenarian from December 28, 2020, to September 23, 2021 (Supplementary Figure S1). The purpose of this study is to provide fresh insights into extreme phenotypes, with a particular focus on immune-inflammatory parameters. To the best of our knowledge, this is the first phenotypic study of the oldest male centenarian showing INFLA score, which is known for evaluating synergistic effect of inflammatory markers [12], and ageing-related immune phenotype indicators (ARIP), recognised as markers of biological age [13]. ARIP ratios are indeed robust indicators that provide a more comprehensive understanding of how T-cell immunity is associated with health than individual T-cell subsets [13]. 

## 2. Materials and Methods

### 2.1. Recruitment

The recruitment of Mr. Turturici was carried out as part of the project “Discovery of molecular, and genetic/epigenetic signatures underlying resistance to age-related diseases and comorbidities (DESIGN)”, funded by the Italian Ministry of Education, University and Research. The Ethic Committee of Palermo University Hospital (Sicily, Italy) approved the study protocol. The study was conducted in accordance with the Declaration of Helsinki and its amendments. Well-trained nutritionists and physicians administered a detailed questionnaire to collect demographic, clinical, and anamnestic data of interest as well as cognitive and functional tests. Our questionnaire includes several sections regarding anamnestic data, cognitive status (mini-mental state examination (MMSE)), activities of daily living (ADL), instrumental activity of daily living (IADL), smoking, alcohol, sleep habits, and health status, such as main pathologies and drugs, geriatric depression scale (GDS), and eating habits [14]. We also investigated the family history. Before enrolment, Mr. Turturici’s daughter provided consent to release the photos and the sensitive data. The semi-supercentenarian underwent venepuncture after a 12 h fasting period in the morning (10 a.m.) on September 16, 2020 (at age of 108 years and 8 months). Blood was collected in specific tubes containing EDTA or no additives.

### 2.2. Molecular Tests

Genomic DNA was extracted from leukocytes using a commercial kit. We genotyped the single-nucleotide polymorphism (SNP) rs2802292 G-allele (G > T) of Forkhead box O3A (FOXO3A) gene using amplification-refractory mutation system–polymerase chain reaction (ARMS-PCR). Three genotypes were analysed: GG, GT, and TT. The size separation was conducted using agarose gel electrophoresis (2%) [14]. To analyse Apolipoprotein (Apo)E polymorphisms, we used the EzWayTM Direct ApoE Genotyping Kit (KOMABIOTECH INC) with standard PCR. The genotype was determined by the combination of three alleles: ε2, ε3, and ε4. The primer mixture of ApoE genes was enabled to perform one-step multiplex PCR. Six genotypes were analysed: ε2/ε2, ε2/ε3, ε3/ε3, ε3/ε4, ε4/ε4, and ε2/ε4. The size separation was conducted using agarose gel electrophoresis (2.5%) [14]. 

#### 2.2.1. Haematological and Haematochemical Parameters 

Whole blood was utilized for automated differential leukocyte counts, and the results were expressed as absolute values using the XN-2000 automated haematology analyser from Sysmex. Lymphocyte subsets were identified through flow cytometry analysis conducted using the FACS Canto II (BD) instrument. These tests were, respectively, carried out at the Department of Laboratory Medicine and the Central Laboratory of Advanced Diagnosis and Biomedical Research at “P. Giaccone” University Hospital in Palermo [11]. Serum levels of immunoglobulins A, G, and M (IgA, IgG, IgM) were also measured using the Roche Diagnostics cobas^®^ 8000 modular analyser with an immunoturbidimetric assay on cobas^®^ c 503 analytical unit at the Department of Laboratory Medicine, where all the other haematochemical tests, as well as serum electrophoresis, were performed [14].

#### 2.2.2. Oxidative and Inflammatory Tests

The evaluation of ox-LDL and uric acid (UA) was carried out as previously described [14]. C-reactive protein (CRP) values were determined using an immunoturbidimetric assay on the cobas^®^ c 503 analytical unit, while interleukin (IL)-6 values were assessed through an immunoassay test utilizing electrochemiluminescence technology on the cobas^®^ e 801 analytical unit [11]. We also assessed the neutrophil/lymphocyte ratio (NLR) and the platelet/lymphocyte ratio (PLR), which have been significantly associated with the occurrence and progression of various inflammatory conditions [15,16]. NLR was calculated by dividing the neutrophil count by the lymphocyte count, while PLR was derived by dividing the platelet count by the lymphocyte count. The INFLA score was calculated for the entire Sicilian population recruited for the DESIGN project (N = 250, age range 19–111 years). The INFLA score was computed by creating 10 tiles for CRP, leukocyte count, platelet count, and NLR values. To generate the 10 tiles, the data for each biomarker were divided into ten groups based on their values, such that the first 10 tiles contained the lowest values, the second contained slightly higher values, and so on, until reaching the tenth 10 tiles containing the highest values. These biomarker 10-tiles were assigned scores ranging from lower levels (from −4 to −1) to higher levels (from +4 to +1), with intermediate values receiving a score of 0. Summing the scores of the four components results in the INFLA score, which ranges between −16 and +16 [12].

#### 2.2.3. ARIP Indicators

Cellular changes in the adaptive immune system accompany the ageing process and contribute to the ageing-related immune phenotype (ARIP), characterized by a decrease in naïve T-cells (TN) and an increase in memory T-cells (TM) [13]. The ARIP indicators, which we analysed based on well-understood age-related changes in T-cell distribution, included the CD4/CD8 ratio, CD4 and CD8 naïve percentages, and the ratios of TN (naïve)/(TCM (central memory) + TEM (effector memory) + TEFF (effector)) (referred to as TN/TM) in CD4+ and CD8+ T-cells. Terminally differentiated effector memory (T_EMRA_) cells are included in TEM [13]. Lymphocyte subsets were identified as previously described [11].

#### 2.2.4. Statistics

No formal statistical analysis has been carried out. For the purpose of comparisons, the reference range values are presented in the various tables, which, unless specified otherwise, are based on a sample of the Sicilian population aged between 18 and 65 years.

## 3. Results

**Figure 1:**
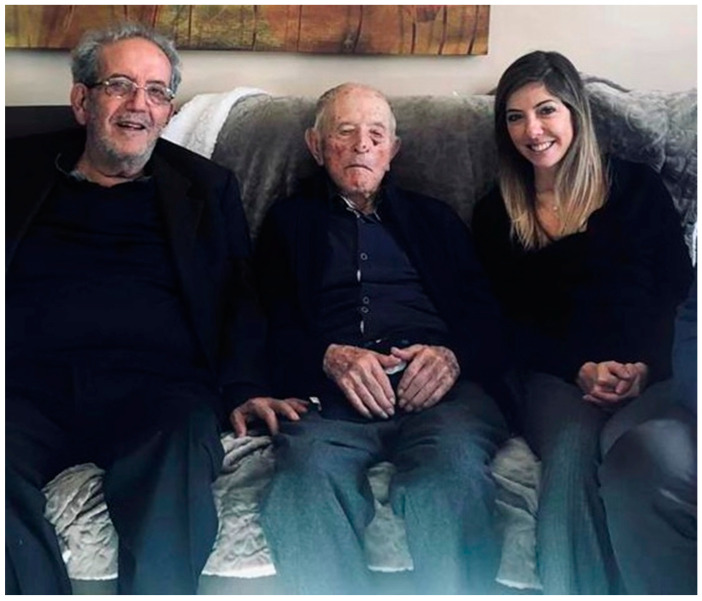
The picture depicts the semi-supercentenarian, Antonino Turturici, with Dr. Anna Aiello and Prof. Calogero Caruso. Photo used with permission.

A.T. was born on 18 January 1912 and passed away on 23 September 2021 in Caltabellotta (Agrigento, Sicily, Italy) (Figure 1). He spent his entire life in Caltabellotta. He completed his military service in Sciacca but did not serve on the front lines due to being the only male in his family. His parents, Pellegrino Turturici and Biagia Ragusa, passed away at the ages of 79 (cause unknown) and 93 (due to a heart attack), respectively. They had five other daughters, four of whom lived to advanced ages (101; 94, who died of colon cancer; 92; and 91), and one who passed away at the age of 80 due to hepatitis. Antonio was married and had two children, a male who lived for only six days and a female who is currently 62 years old and dealing with hypertension. Antonio himself completed primary school, receiving 5 years of education. As a landowner, he had a consistent income throughout his life, which allowed him to live without financial difficulties. He even travelled extensively within Italy and abroad until the age of 88. After his wife’s passing in 2017, he continued to reside in his own home, receiving care from his daughter. 

He never smoked, typically slept around 6 h per night, and enjoyed a few half glasses of wine at the table. He was under treatment for various health conditions, including lansoprazole for managing oesophageal reflux, diuretics and digoxin to address heart failure, and choline alfoscerate as a dietary supplement. In 2000, he experienced melena, likely due to antiplatelet agents. Over the past few years, he had several falls, with the most recent incident occurring in June 2020 when he suffered a fractured femur. He successfully underwent surgery and was discharged after a four-day hospital stay without requiring any transfusions.

With a height of 1 m and 58 centimetres and a weight of 62 kg, his body mass index (BMI) was calculated to be 24.8, and his waist circumference measured 85 centimetres. Notably, his blood pressure was within a healthy range at 110/60 mmHg, although his heart rate was somewhat elevated (105 beats per minute).

The administration of the GDS indicated an absence of depression. Consequently, he maintained an optimistic outlook on life, which he regarded as the best possible. The MMSE was not administered due to both visual and hearing impairments. The assessment of activities of daily living (AD), encompassing personal hygiene, dressing, bathroom use/continence, walking, and eating, revealed a need for assistance in meeting these basic physical requirements for approximately eight years. Additionally, instrumental activities of daily living (IADL, such as food preparation, financial management, house cleaning, phone use, and medication responsibility) revealed a prolonged inability to perform these complex household tasks.

Regarding eating habits during childhood (up to the age of 15), A.T.’s diet adhered fairly closely to the Mediterranean diet (MedDiet). This was evident through a limited consumption of red meat (once a week) and the daily intake of fruits or vegetables twice, as well as bread or pasta. Eggs were consumed two to three times a week, while fish and sweets made occasional appearances in the diet. Legumes, particularly fava beans, were regularly consumed, especially during the winter and spring.

As for current eating habits, there was limited adherence to the MedDiet, except for the consumption of grains like pasta, extra virgin olive oil, milk, fruits, and vegetables, which were consumed once a day. Legumes were consumed two to three times a week. Conversely, there was a high consumption of sweets (such as cookies, small pastries, and sugar) twice a day and of red meat (in pureed form) once a day.

Table 1 presents the ApoE and FOXO3A data. Regarding ApoE, he did not possess either the favourable ε2 allele or the detrimental ε4 allele. Concerning the FOXO3A gene, A.T. did not carry the longevity-associated G SNP [14,17]. Table 2 illustrates how the haematological values, including the main lymphocyte subsets, fell within the laboratory reference range. Concerning Ig levels, IgG and IgM fell within the laboratory reference range, but IgA levels were higher.

Regarding the haematochemical parameters (Table 3), the endocrine and iron markers were within the laboratory reference range. Lipid markers also fell within the laboratory reference range, although HDL values were at the lower limits. A different situation arose with the liver markers, as albumin and total protein values were reduced. However, transaminase, GGT, and bilirubin values were within the reference range. Concerning the catabolic parameters, creatinine and urea were within the laboratory reference range. When considering bone markers, ALP values were higher than the reference range, and the vitamin D values were extremely low.

**Figure 2:**
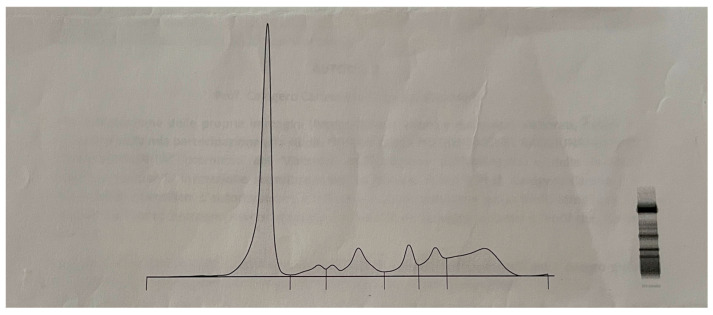
Serum protein electrophoresis percentages (reference range in parentheses). Albumin, 51.8% (54.5–65.0); α1 globulins, 3.4% (3.1–6.0); α2 globulins, 10.8% (7.1–11.8); β1 globulins, 7.1 (5.0–7.2); β2 globulins, 8.0 (3.2–6.5); γ globulins, 18.9 (10.5–18.8).

Figure 2, then, illustrates the serum electrophoresis pattern that confirms the haematological and haematochemical data. In fact, the electrophoretic pattern reveals a decrease in the percentage of albumin, an increase in β2 globulins, and a slight increase in the γ globulin.

As shown in Table 4 (oxidative and inflammatory tests), the IL-6 values and INFLA scores exceeded the control values, whereas the other parameters are within the reference range. Table 5 presents the ARIP indicators. Regarding ARIP, CD4+ naïve, CD4/CD8 ratio, and TN/TM (CD4) ratio values fell within the laboratory reference range, whereas CD8+ naïve and TN/TM (CD8) ratio were outside the reference range.

## 4. Discussion

It is beyond doubt that A.T. belonged to a long-lived family. However, it is important to clarify that in the human species, family ties do not necessarily only imply a genetic connection but can also involve shared environment and lifestyle. As for the genetics, it should not be surprising that in Sicilian centenarians including A.T., there is no association with the FOXO3A and APOE genes. As elaborated below, it is essential to consider the dynamic interplay between genetic variations and environmental factors in the development of individual differences in health and in longevity [18,19]. On the other hand, emerging evidence suggests that multiple rare and protective variants, varying among different long-lived families, are associated both with healthy ageing and extreme longevity [20].

In our Sicilian studies [14,17], longevity concerns individuals living in small towns or villages located in the mountainous regions of inland Sicily, away from major polluted cities. In these villages, the older population has experienced different working conditions compared to those in large cities, as well as distinct lifestyles, including reduced smoking and alcohol consumption and adherence to the MedDiet during childhood. Notably, a physically active lifestyle, involving regular outdoor activities for commuting to work, is common in these areas due to the generally steep terrain, believed to contribute to extended and intense physical activity. This, in turn, improves the cardiorespiratory and immune capacity of the inhabitants [21,22]. Furthermore, in these small towns and villages, people have better access to family support and social networks, resulting in better healthcare and lower mortality rates, particularly among those with female offspring. It is intriguing that A.T. spent his entire life in Caltabellotta, a mountainous village (949 m above sea level) with a population of 3314, living with his family and receiving care from his daughter. All these factors have contributed to his optimistic outlook on life, which, in turn, may have played a role in his extreme longevity [23].

Regarding his diet, it was rich in bioactive Mediterranean foods like fruits and legumes, although it did not strictly adhere to the traditional MedDiet. Starting from the 1960s, the consumption of meat, fish, fats, and sugars significantly increased in the southern regions of Italy, while the consumption of bread, pasta, cereals, vegetables, and olive oil began to decrease. During this nutritional transition, there was likely a substantial change in A.T.′s diet, as in the rest of Italy [14]. On the other hand, our study of Sicilian centenarians confirms their adherence to the MedDiet at a young age due to food scarcity rather than choice. Nutritional options were closely tied to seasons, and the quantity of food was sufficient but never excessive. The diet was quite monotonous and often centred around legumes as the primary choice [14,17]. All these factors could potentially have influenced an individual’s ability, including A.T., to achieve extreme longevity through epigenetic modifications [24,25].

In relation to BMI, usually, centenarians are underweight; however, A.T. had normal weight. His BMI and waist circumference were both within the normal range. These findings are interesting considering that underweight and overweight conditions, as well as reductions or increases in BMI, are unfavourable for longevity [14].

The ApoEε4 allele, known for its pro-inflammatory properties, poses a risk factor for the development of both Alzheimer′s disease and cardiovascular diseases, exerting a detrimental influence on longevity, whereas the presence of the ε2 allele promotes longevity. The ε3 allele, on the other hand, is considered neutral [26]. Our survey on Sicilian centenarians failed to establish a correlation between ε2 and longevity, although the prevalence of the ε4 allele was lower compared to that in the general population [14]. Accordingly, our semi-supercentenarian carried the genotype ε3/ε3. These findings can be explained by a study indicating that the protective impact of ε2 is less pronounced in populations originating from Southern Italy and that ε4 lacks a harmful effect, highlighting the pivotal role of the MedDiet adhered to by the centenarians during their early years [26].

The transcription factor FOXO3A performs crucial regulatory functions in insulin-like growth factor signalling. Activation of this pathway by a diet abundant in animal proteins and refined sugars curtails its transcription, ultimately promoting unsuccessful ageing [27]. Investigations conducted across several populations have revealed an association between the SNP rs2802292 (allele G) and longevity, most likely due to escalated FOXO3A expression implicated in homeostatic responses [27]. In contrast, our semi-supercentenarian was of the TT genotype. However, within the Sicilian centenarians, this longevity-linked association was not apparent, plausibly attributed to the aforementioned reason, i.e., this generation adhered closely to the MedDiet during their early years.

Recently, Caruso et al. [20] reviewed several studies on the genetics of longevity. The results reveal that despite efforts and new technologies, only two genes, APOE and FOXO3A, which we have studied in this report, have consistently shown associations with longevity in most studies. This is due to the dynamic interaction between genetic and environmental variations in shaping individual differences in health and longevity [18]. However, it is believed that genetics contribute 33% to women and 48% to men in reaching the age of one hundred years. In various studies, the effect sizes are not substantial, suggesting that many genes with modest effects are involved in the genetic component of longevity, as is the case with all multifactorial traits [28,29,30,31,32]. Therefore, it is not surprising that associations replicated by GWAS with common variants related to longevity are limited, largely because they aggregate diverse populations, missing the ‘ecological’ dimension of longevity. Rare variants are numerous and likely vary in different populations to such an extent that it has been proposed that studies involving families with several long-lived individuals hold particular promise for their discovery [33].

All haematological parameters, including lymphocyte subsets, fell within the reference ranges of adult people. Furthermore, the values of the lymphocyte subsets were higher than or equal to the average values of individuals over ninety years old.

Regarding the immunological serological tests, IgG and IgM were within the reference range, while IgA levels were elevated. In fact, the IgA values aligned with values observed in a previous study [34] involving 11 centenarians (99–108 years old). We did not detect any suspicious bands in serum electrophoresis, but second-level investigations for detecting them were not performed, and the presence of an inconspicuous monoclonal component cannot be ruled out. It is important to highlight that there is emerging evidence suggesting an inflammatory role for IgA [35].

In terms of endocrine markers, the low levels of the HOMA index clearly indicate good glucose control, a reduced risk of type 2 diabetes, and a lower likelihood of associated health complications [36]. A.T. exhibited decreased levels of albumin and total proteins, consistent with findings from various centenarian studies [14,37]. While this datum has been used as a biochemical indicator of nutritional status, it is more likely that in the oldest people, including A.T., the low levels reflect the chronic inflammatory state of advanced age, as albumin is a negative acute-phase protein. All iron markers were found to be within the laboratory reference range, despite the expected decrease in serum iron levels due to the chronic inflammatory state associated with ageing [38].

The lipid marker data we obtained from the Sicilian centenarians were largely consistent with existing findings in the literature [14]. The literature data suggest that the levels of total cholesterol, LDL, and HDL among centenarians do not significantly differ from those observed in their older adult counterparts, and their triglyceride levels are comparable to those of healthy older adults [37]. Nevertheless, the lipid measurements of our semi-supercentenarian virtually fell within the reference range provided by the laboratory.

Interestingly, not only were his magnesium values within the expected reference range [14], but his calcium levels were also maintained within the range despite his apparent deficiency in vitamin D, possibly due to inadequate dietary intake or more likely, limited exposure to sunlight [14,39]. Regarding the increase in ALP, osteoporosis is often associated with an imbalance between bone resorption and bone formation. Osteoblasts are the primary producers of ALP. In individuals with osteoporosis, there can be an alteration in bone turnover, leading to changes in ALP levels. Elevated ALP levels might suggest increased bone remodelling as the body attempts to restore bone density [40].

It is worth noting that the urea and creatinine values remained within the reference range, which contrasts with the anticipated increase associated with the age-related gradual decline in kidney function [41].

UA is the end product of purine metabolism. The role of UA is contentious, as it has been reported to increase oxidative stress, while other studies suggest that UA acts as a scavenger of reactive oxygen species, exerting an antioxidant effect [42,43]. Notably, in our recent proteomic study, several proteins exhibited correlations with both age and UA, potentially forming a distinctive signature for healthy ageing [44]. However, the UA levels as well as the oxLDL levels fell within the reference ranges. These findings align with the majority of the literature that reports a decrease in oxidative stress in the long-lived. In fact, in most studies, centenarians have demonstrated lower levels of lipid peroxides and higher plasma levels of the antioxidant vitamin E compared to those of older controls, suggesting that they might be better equipped to counter oxidative stress [45].

In summary, the results of the laboratory analyses conducted, as well as the clinical history and interview data (which allowed us to appreciate his optimistic outlook on life), when compared to the results of our recent study on Sicilian centenarians (aged 100–111 years) [14], demonstrate a state of health that we can consider excellent, considering his age. This is true even in the presence of osteoporosis, which, as it turned out, was quite common among the Sicilian centenarians studied [14].

In line with prior studies [11,22,45,46,47,48], we observed a rise in the inflammatory markers of IL-6 and INFLA score in our oldest male centenarian. IL-6 is known to elevate in response to inflammatory triggers [49]. The INFLA score helps assess the possible synergistic effect of inflammatory biomarkers that can produce multi-collinearity when simultaneously studied, ignoring the variability presented by the differences in units, mean intakes, and biological actions [12]. The increase in β2-globulins also indicates the chronic inflammatory state of A.T. In our comprehensive immunological investigation carried out among Sicilians, we noted a progressive rise in inflammatory markers with advancing age. Nevertheless, some of the oldest centenarians displayed inflammatory markers similar to those found in younger counterparts [11]. However, this was not the case with A.T., whose levels appeared elevated. However, the detrimental impacts of inflamm-ageing among centenarians could potentially be mitigated through various mechanisms [50,51,52]. On a different note, considering that A.T. passed away just a year after the blood sample was obtained, these elevated levels align with the notion that inflammation should be a predictive factor for centenarian mortality [17,45].

An immune phenotype related to ageing has been defined as a decrease in naive T-cells compared to the accumulation of memory T-cells. These age-related immunophenotypic changes in T-cell subsets reduce immune protection against pathogens and new viral infections and decrease vaccine responsiveness [22]. A recent study discovered two ARIP indicators of T-cells to predict chronic diseases and mortality. Specifically, a higher ratio of CD4+TN to CD4+TM (CD4+TN/TM) was inversely correlated with biological aging, multimorbidity, and mortality. Thus, ARIP ratios are robust indicators that provide a better understanding of how T-cell immunity is associated with health compared to individual T-cell subsets [13]. Furthermore, it is interesting that a recent study provided evidence that personality is related to ARIP. Higher conscientiousness and, to a lesser extent, greater extraversion may be protective against age-related immunophenotypic change [53]. Thus, it is very intriguing that the semi-supercentenarian displayed ARIP marker values such as naive CD4+ cells, the CD4+/CD8+ ratio, and CD4+TN/TM ratio within the range of young adult individuals. This suggests that his immune system had a younger biological age compared to his chronological age [13]. Regarding the levels of naive CD8 cells and the TN/TM ratio in CD8 cells that were below the reference range, it is known that such levels are influenced by chronic Cytomegalovirus (CMV) infection rather than biological or chronological age. A.T., like all ultra-nonagenarians in Sicily, was CMV-positive, and the virus, even in a latent state, continually stimulates the immune system [11,22,54].

The potential role of the immune system in contributing to extreme longevity is a subject that lacks universal acceptance. This is due to the fact that centenarians display changes in their immune systems associated with ageing, known as immunosenescence [11,54].

Recent findings on lymphocyte subsets in semi-supercentenarians and supercentenarians [11,54,55,56,57,58] suggest that immune system ageing should be considered as a specific adaptation that enables the oldest centenarians to successfully cope with a lifetime of antigenic challenges and achieve extreme longevity. The data from this study on ARIP, although limited to the analysis of only one semi-supercentenarian, demonstrate a biological age of his immune system younger than his chronological age, which strengthens the idea that the immune system plays a role in promoting extreme longevity. In fact, analysing the ARIP indicators of the 58 subjects participating in the study by Ligotti et al. [11], which included A.T. and seven of the oldest centenarians, there is a trend for a better biological profile in semi- and supercentenarians compared to people aged between 95 and 104 years. However, the data did not allow us to draw statistically significant conclusions due to their considerable heterogeneity and relatively small sample size (unpublished observations).

## 5. Conclusions

While exploring the role of the immune system in attaining extreme longevity, it is essential to acknowledge that the immune system of older people has been subject to more extensive and in-depth research compared to other bodily systems and organs, primarily due to its amenability to ex vivo studies. On the other hand, immune ageing and the preservation of a relatively robust immune response may only represent components of the overall deterioration or, conversely, the general well-functioning of the organism, which is regulated by factors beyond the immune system (such as the brain and the endocrine system governed by the brain). Nevertheless, both immune ageing and good immune function could play pivotal roles in the processes of ageing and longevity, respectively. Consequently, the immune system assumes a significantly influential role in the quest for longevity, yet this does not preclude the involvement of other bodily systems or organs.

However, present findings are consistent with the hypothesis that both semi-supercentenarians and supercentenarians exhibit increasing relative resistance to age-related diseases. They approach the limits of human functional reserve to successfully combat acute causes of death [6,59,60].

## Figures and Tables

**Table 1:** Molecular tests.

Variable	A.T.	Young Adults N = 29	Centenarians N = 22
APOE	N alleles	N alleles	N alleles
ε3	2	5	4
ε2		49	39
ε4		4	1
FOXO3A rs2802292	N alleles	N alleles	N alleles
G		25	17
T	2	33	27

A.T. = Antonino Turturici; N = Number; Young Adults (18–39 years); Centenarians (100–111 years).

**Table 2:** Haematological parameters.

Variable (Unit)	Values	Laboratory Reference Range Value
Red Blood Cells (10^6^ µL)	4.39	4.20–5.50
Haemoglobin (g/dL)	13.70	12.00–18.00
Platelets (10^3^ µL)	230	150–450
Leukocytes (10^3^ µL)	7.61	4.00–11.00
Neutrophils (10^3^ µL)	4.12	2.00–8.00
Eosinophils (10^3^ µL)	0.37	0.00–0.80
Basophils (10^3^ µL)	0.05	0.00–0.20
Monocytes (10^3^ µL)	0.94	0.16–1.00
Lymphocytes (10^3^ µL)	2.12	1.00–5.00
CD3 (10^3^/μL)	1.25	0.81–2.13
CD4 (10^3^/μL)	0.70	0.02–1.88
CD8 (10^3^/μL)	0.51	0.06–0.74
IgG (mg/dL)	1145	700–1600
IgA(mg/dL)	*551*	70–400
IgM (mg/dL)	94.2	40–230

Values out of range are in bold, italic, and underlined.

**Table 3:** Haematochemical parameters.

Variable (Unit)	Values	Laboratory Reference Range Value
Endocrine Markers		
TSH (µIU/mL)	0.56	0.27–4.20
FT3 (pg/mL)	2.65	2.00–4.40
FT4 (ng/dL)	1.31	0.93–1.70
Insulin (µU/mL)	3.28	2.60–24.90
HOMA Index	0.56	0.47–3.19
Glycaemia (mg/dL)	*69*	70–100
Liver Markers		
ALT (U/L)	13	<41
AST (U/L)	23	<40
GGT (U/L)	53	8–61
Bilirubin (mg/dL)	0.85	<1.20
Albumin (g/L)	*33.4*	38–48
Proteins (g/L)	*58.6*	66–87
Iron Markers		
Iron (µg/dL)	85	37–145
Ferritin (ng/mL)	91	15–400
Transferrin (mg/dL)	266	200–360
Lipid Markers		
Total Cholesterol (mg/dL)	133	<200
LDL (mg/dL)	*64.4*	>65
HDL (mg/dL)	56	>50
Triglycerides (mg/dL)	63	<200
Bone Markers		
Osteocalcin ng/mL	33.4	14.00–46.00
ALP (U/L)	*175*	40–129
Calcium (mg/dL)	8.61	8.40–10.20
Magnesium (mg/dL)	2.13	1.60–2.60
Vitamin D (ng/mL)	*3.75*	(>30)
Catabolic Parameters		
Creatinine (mg/dL)	1.01	0.5–1.2
Urea (mg/dL)	33.5	16.8–48.5

Values out of range are in bold, italic, and underlined. TSH, thyroid-stimulating hormone; T3, triiodothyronine; T4, thyroxine; HOMA, homeostasis model assessment; ALT, alanine transaminase; AST, aspartate transaminase; ALP, alkaline phosphatase; GGT, gamma glutamyl transferase; LDL, low-density lipoprotein; HDL, high-density lipoprotein.

**Table 4:** Oxidative and inflammatory tests.

Variable (unit)	Values	Laboratory Reference Range Values
LDL Ox (mIU/mL)	47	*44.6–87.3*
Uric Acid (mg/dL)	6.0	2.4–7.0
CRP (mg/dL)	3.37	<5 mg/dL
IL-6 (pg/mL)	*17.8*	<7 pg/ml
NLR	1.95	0.92–2.84
PLR	108.02	074.71–193.34
INFLA score	*8*	−1.25 *

Values out of range are in bold, italic, and underlined; NLR, neutrophil/lymphocyte ratio; PLR, platelet/lymphocyte ratio; * mean of the scores of 99 adults (19–65 years).

**Table 5:** ARIP indicators.

Variable (Unit)	Values	Laboratory Reference Range Values
CD4+ Naive (CD45RA + CD27+) (%)	24.0	4–57
CD8+ Naive (CD45RA + CD27+) (%)	*9*	10–78
CD4/CD8	1.37	0.85–5.04
TN/TM (CD4)	0.32	0.05–1.35
TN/TM (CD8)	*0.10*	0.11–3.48

Values out of range are in bold, italic, and underlined. TN T naïve; TM T memory.

## Data Availability

The data that support the findings of this study are available from the corresponding author, upon reasonable request.

## Supplementary Materials

The following supporting information can be downloaded at: https://www.mdpi.com/article/10.3390/jcm12247591/s1. Supplementary Figure S1. Recruitment and age validation of Mr. Antonino Turturici.
