# Supplementary material for: The Phenotypic Characterization of the Oldest Italian Man from December 28, 2020, to September 23, 2021, A.T., Strengthens the Idea That the Immune System can Play a Key Role in the Attainment of Extreme Longevity
Source: J Clin Med. 2023 Dec 9;12(24):7591. doi: 10.3390/jcm12247591 (PMC10744028; doi:10.3390/jcm12247591)
Supplement: Supplementary file 1 [file jcm-12-07591-s001.zip › jcm-2630143-supplementary.pdf]

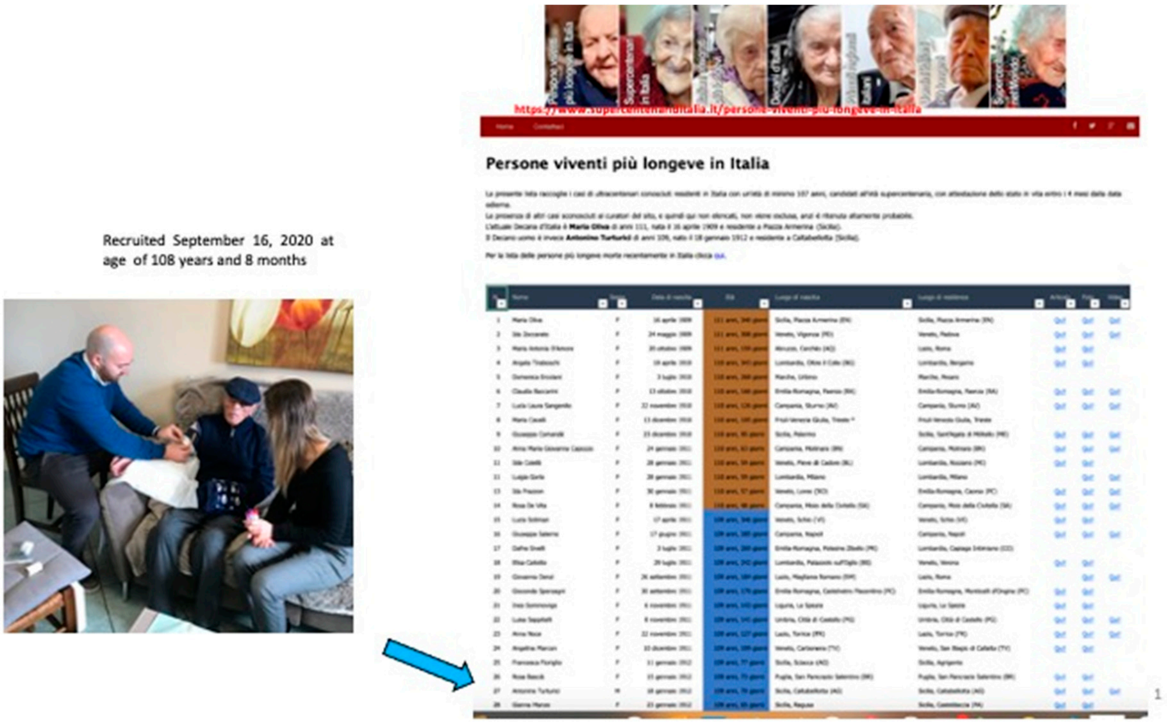

Supplementary Figure S1. Recruitment and age validation of Mr. Antonino Turturici. The website https://www.supercentenariditalia.it/persone-viventi-piu-longeve-in-italia, curated by Dr. De Lucchi, includes the names of known supercentenarians residing in Italy who are at least 107 years old, with proof of their current status. All cases are validated through in-person or remote verification of the individual's identity card.
